# Unusual traits of *cis* and *trans*-2,3-dibromo-1,1-dimethylindane on the way from 1,1-dimethylindene to 2-bromo-, 3-bromo-, and 2,3-dibromo-1,1-dimethylindene

**DOI:** 10.3762/bjoc.12.113

**Published:** 2016-06-10

**Authors:** Rudolf Knorr, David S Stephenson, Ernst Lattke, Petra Böhrer, Jakob Ruhdorfer

**Affiliations:** 1 Department Chemie, Ludwig-Maximilians-Universität München, Butenandtstrasse 5–13 (Haus F), 81377 München, Germany

**Keywords:** base-free dehydrobromination, *cis*/*trans* stereochemistry, five-membered ring conformation, indenes, NMR couplings

## Abstract

Do not rely on the widely accepted rule that vicinal, sp^3^-positioned protons in cyclopentene moieties should always have more positive ^3^*J* NMR coupling constants for the *cis* than for the *trans* arrangement: Unrecognized exceptions might misguide one to wrong stereochemical assignments and thence to erroneous mechanistic conclusions. We show here that two structurally innocent-looking 2,3-dibromo-1,1-dimethylindanes violate the rule by means of their values of ^3^*J*(cis) = 6.1 Hz and ^3^*J*(trans) = 8.4 Hz. The stereoselective formation of the trans diastereomer from 1,1-dimethylindene was improved with the tribromide anion (Br_3_^−^) as the brominating agent in place of elemental bromine; the ensuing, regiospecific HBr elimination afforded 3-bromo-1,1-dimethylindene. The addition of elemental bromine to the latter compound, followed by thermal HBr elimination, furnished 2,3-dibromo-1,1-dimethylindene, whose Br/Li interchange reaction, precipitation, and subsequent protolysis yielded only 2-bromo-1,1-dimethylindene.

## Introduction

The basic mechanistic features of competing suprafacial and antarafacial additions of elemental bromine to an olefin are reasonably well established [1–3]. With indene as a cyclic olefin, the major product *trans*-1,2-dibromoindane was formed through antarafacial addition; the accompanying *cis* diastereomer resulted through the suprafacial bromine addition and rarely [4] exceeded 30% of the diastereomeric *trans*/*cis* product mixture. For the *cis*/*trans* assignments, common wisdom [5] commends the simple criterion that three-bond NMR coupling constants (^3^*J*_H,H_) should be more positive for a *cis* than for a *trans* relationship of the vicinal, sp^3^-positioned protons in five-membered rings. Indeed, *cis*-1,2-dibromoindane displayed ^3^*J*(1-H,2-H) = 5.0 Hz, whereas the *trans* diastereomer exhibited ^3^*J*(1-H,2-H) = 1.3 Hz [6]. However, we report here on the closely related *trans* diastereomer of 2,3-dibromo-1,1-dimethylindane (*trans*-**1**) whose grossly deviating value of ^3^*J*(2-H,3-H) = 8.4 Hz violates the above ^3^*J*_H,H_ rule.

## Results and Discussion

**Scheme 1:**
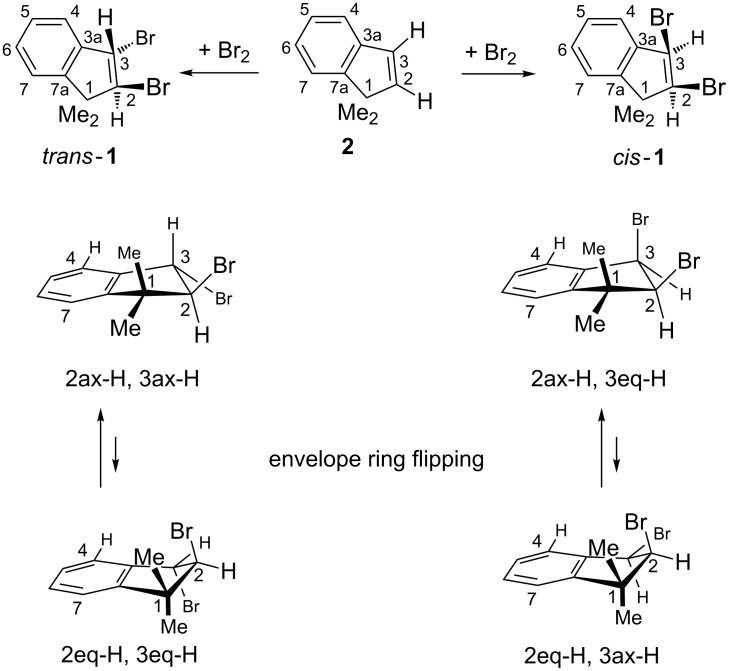
Bromine adducts (*trans*-**1** and *cis*-**1**) of 1,1-dimethylindene (**2**) and their envelope conformations: ax = pseudoaxial, eq = pseudoequatorial; Me = methyl.

The addition of elemental bromine to 1,1-dimethylindene (**2**, see Supporting Information File 1) in CCl_4_ as the solvent afforded a 7:3 mixture of *trans*-**1** and *cis*-**1** (Scheme 1). A more useful 9:1 mixture was obtained through the slow titration of a well-stirred chloroform solution of equimolar amounts of **2** and tetraethylammonium bromide with elemental bromine in chloroform. Such a higher *trans* selectivity is typical of the very rapidly [1] formed tribromide anion (Br_3_^−^) as the reactive species.

What kind of evidence supports our stereochemical assignments of *trans*-**1** and *cis*-**1**? Although the three-bond NMR coupling constant ^3^*J*(2-H,3-H) = 6.1 Hz of *cis*-**1** is close to normal, *trans*-**1** exhibits the abnormally high value of 8.4 Hz (compare that with 1.3 Hz for *trans*-1,2-dibromoindane [6]), violating the commonly accepted ^3^*J*_H,H_ rule that was mentioned in the Introduction. A more reliable criterion may be visualized from the envelope shape (expected puckering ca. 22–33°) of the cyclopentene parts of **1** (lines 2 and 3 of Scheme 1): the distance between two vicinal, sp^3^-positioned protons in indanes clearly must be substantially longer for the pseudodiaxial (2ax-H,3ax-H) *trans* than for the pseudoaxial/pseudoequatorial (2ax-H,3eq-H) *cis* interproton relationships. Such distances may be estimated through nuclear magnetic Overhauser enhancements (NOE). As expected if *trans*-**1** populates predominantly the 2ax-H,3ax-H conformation shown in Scheme 1, our one-dimensional NOE difference experiments revealed an approximately eight-fold stronger enhancement of the 3-H doublet signal of *cis*-**1** than of *trans*-**1** on irradiation of the almost coincident two 2-H NMR doublets of the two diastereomers; this established our assignments. In addition, a two-dimensional NOESY experiment displayed a cross-peak between 3-H and one of the two 1-C*H*_3_ signals of *trans*-**1**. The necessary short distance of the involved protons can be traced to a 1,3-pseudodiaxial arrangement of 3-H and one of the methyl groups in the 2ax-H,3ax-H conformation. Thus, a close to 160° torsional relationship between the C(2)–H and C(3)–H bonds gives rise to the surprisingly high value of ^3^*J*(2-H,3-H) = 8.4 Hz [7–8]. The hyperconjugative interaction of this pseudoaxial C(3)–H bond with the aromatic π system causes a long-range (hence weak) magnetic coupling (*J* = 0.7 Hz) between 3-H and (presumably) 4-H in *trans*-**1** (but not found in *cis*-**1**). The different conformational preference of *trans*-**1** as compared with the methyl-free *trans*-1,2-dibromoindene (^3^*J* = 1.3 Hz [6]) may obviously be ascribed to the two 1-Me groups [9]. In accord with the pseudoaxial C(3)–H bond, HMBC (hetero multiple bond correlation) cross peaks of 3-H in *trans*-**1** were absent with both C-1 and C-7a. On the other hand, the corresponding cross peaks were strong in *cis*-**1** between 3eq-H and both C-1 and C-7a, which indicated a significant ^3^*J*_C,H_ NMR coupling via the intervening single bonds in their roughly coplanar arrangement shown in Scheme 1. It may also be noticed that the small C(2)–H/C(3)–H torsional angles in either one of the two *cis*-**1** conformations are of similar sizes and do not permit a conformational differentiation. On the other hand, the close to 90° torsional angle between 3-H and 2-H in the 2eq-H,3eq-H conformation of *trans*-**1** would imply an almost vanishing ^3^*J*_H,H_ value, in contrast with the observed value of 8.4 Hz that is explained by the predominant 2ax-H,3ax-H conformation.

**Scheme 2:**
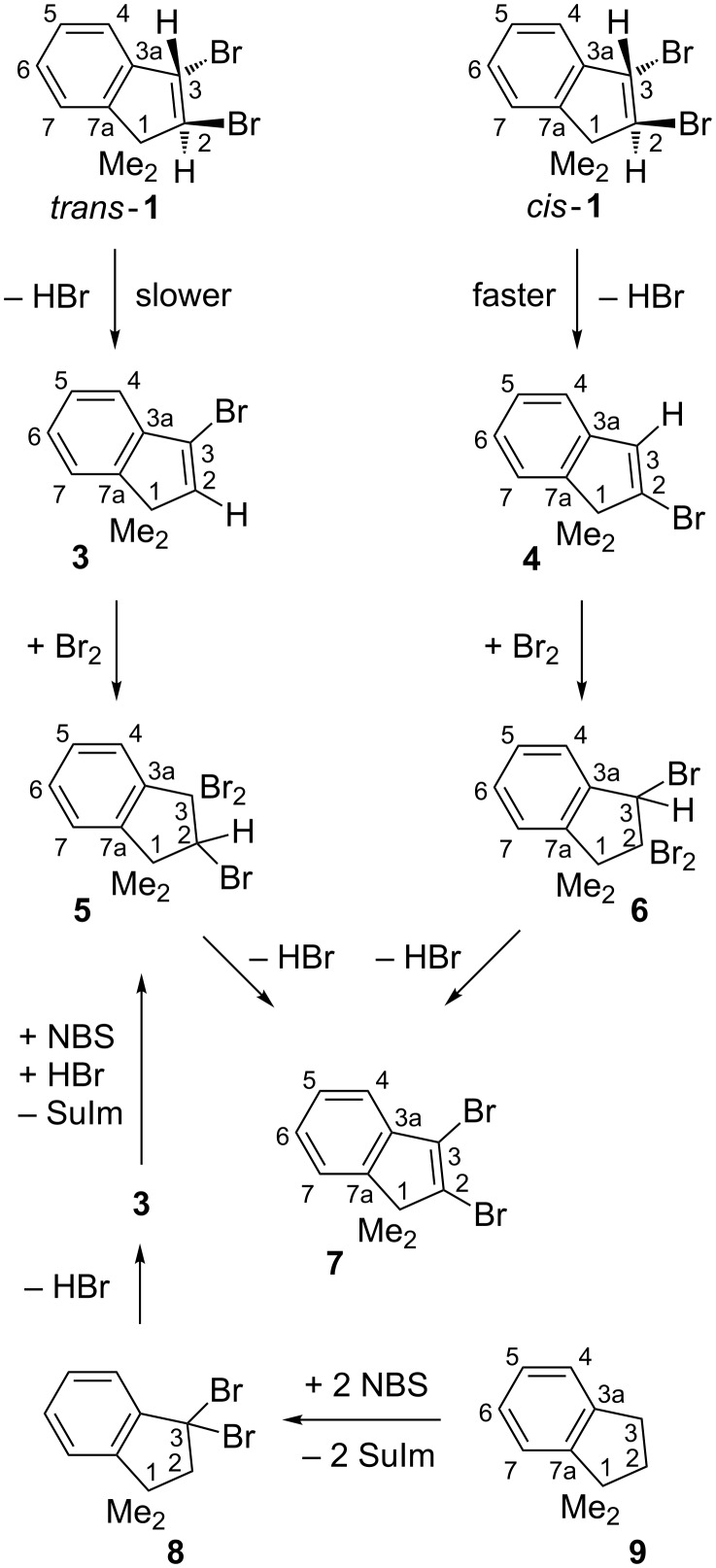
The ways to 2-bromo- (**4**), 3-bromo- (**3**), and 2,3-dibromo-1,1-dimethylindene (**7**); SuIm = succinimide, NBS = *N*-bromosuccinimide, Me = methyl.

Distillation of the *trans*/*cis* product mixture led to some enrichment of the thermally more stable diastereomer *trans*-**1** due to the “base-free” HBr elimination from *cis*-**1** with formation of 2-bromo-1,1-dimethylindene (**4** in Scheme 2) [10]. We observed a less distinct kinetic preference in the corresponding base-induced processes: In di(2-methoxyethyl) ether (diglyme) as the solvent, a substoichiometric amount of KO*t*-Bu (potassium *tert*-butoxide) reacted faster with *cis*-**1** than with *trans*-**1** by a factor of roughly 9 at room temperature (rt). With an excess (>2 equiv) of KO*t*-Bu, both of these weakly exothermic HBr elimination reactions were completed within less than 30 min. With KO*t*-Bu (at rt) or KOEt (at 50 °C) but not with NEt_3_ (no reaction at rt), the exclusive formation of 3-bromo-1,1-dimethylindene (**3**) from *trans*-**1** and of **4** from *cis*-**1** became evident through the similarity of the emerging **3**/**4** ratios as compared with the *trans*-**1**/*cis*-**1** ratios in the employed mixtures [11]. This regiospecificity is readily understandable since *trans*-**1** has no anti relationship of vicinal Br and H available (Scheme 1), whereas each *cis*-**1** conformer holds a pseudodiaxial, vicinal Br/H anti relationship and hence is (presumably) able to react somewhat faster.

We abstained from separating *trans*-**1** and *cis*-**1** since the availability of two different mixtures facilitated the NMR assignments and because any *cis*-**1**/*trans*-**1** mixture or the ensuing **3**/**4** mixtures may be used for preparing 2,3-dibromo-1,1-dimethylindene (**7**). Both **3** and **4** can add elemental bromine and the adducts (**5** or **6**, respectively) eliminated HBr either spontaneously (**5**) or in the presence of KO*t*-Bu or NEt_3_ (**6**) at rt to furnish the same product **7**. We found through NOESY and HMBC analyses that the thermally more stable tribromide **6** populates the conformation which has a pseudoaxial 3-H (and hence a pseudoequatorial 3-Br) orientation (like *trans*-**1**). Therefore, the unusually high thermolability of **5** seemed to be due to the unavoidable presence of one pseudoaxial C(3)–Br bond [12]. If so, the hitherto unknown 3,3-dibromo-1,1-dimethylindane (**8**) might also be prone to such thermal HBr elimination. For comparison, treatment of 1,1-dimethylindane (**9**) with one equivalent of *N*-bromosuccinimide (NBS) furnished the expected 3-bromo-1,1-dimethylindane (see Supporting Information File 1), which decomposed already on distillation to yield 1,1-dimethylindene (**2**). With two equivalents of NBS, however, **9** afforded a mixture of **3** and **7** through the following sequence in the bottom part of Scheme 2: **9** → **8** → **3** → **5** → **7**. Under these conditions, the spontaneous HBr elimination from **8** had produced **3**, which added Br_2_ to generate the thermolabile tribromide **5**, whose HBr elimination gave **7**; the required Br_2_ was visible in the weakly brownish gas phase and had been provided through the well-known reaction of HBr with the accompanying NBS. Consequently, three equivalents of NBS would be necessary for obtaining **7** in a maximum yield. This encouraged us to reflux **9** in CCl_4_ with NBS (4 equiv), which furnished mainly **7** along with succinimide (3.6 equiv) and remnant NBS (0.4 equiv). As a side-reaction, the slower thermal HBr elimination from the intermediate 3-bromo-1,1-dimethylindane (see Supporting Information File 1) generated 1,1-dimethylindene (**2)**, whose in situ bromination furnished **1** (ca. 1%) in a *trans*/*cis* ratio of ca. 3:2. Since both dibromides *trans*-**1** and *cis*-**1** were stable under the reaction conditions and would distil together with **7**, they were destroyed through HBr elimination by KO*t*-Bu (or KOH in EtOH) to produce the monobromides **3** and **4**. It may be noticed that **4** (from *cis*-**1**) cannot have been an intermediate in the initial step of the above “base-free” conversion of **9** to **7**, since **4** would generate the thermally stable tribromide **6** that was not detected in the initial product mixture of **1** and **7**.

**Scheme 3:**
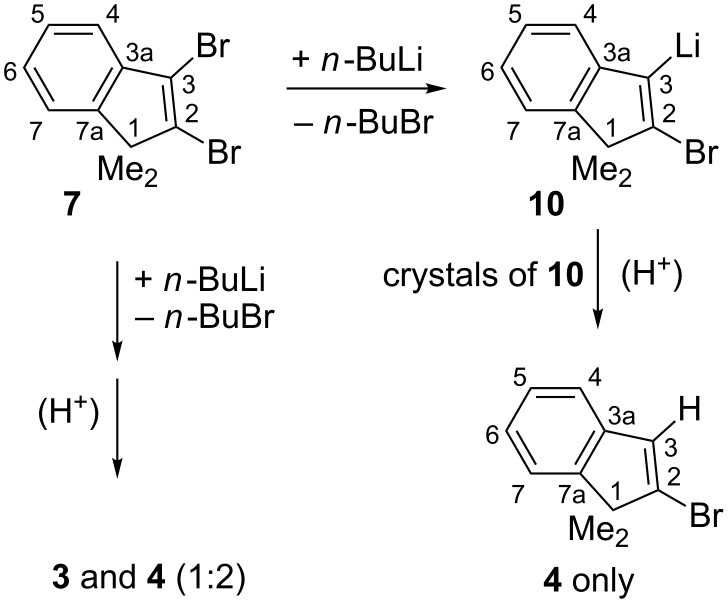
Unsolvated 2-bromo-3-lithio-1,1-dimethylindene (**10**) precipitated from the reaction mixture of **7** with *n*-BuLi in hexane; after purification, **10** provided **4** only; Me = methyl.

For practical purposes, **7** may be useful as an alternative starting material in place of 2,3-diiodo-1,1-dimethylindene that had been employed [13–14] in cross-coupling studies. We actually used crude **7** as follows for a first specific route to 2-bromo-1,1-dimethylindene (**4**). The rapid Br/Li interchange reaction of **7** in hexane as the solvent with *n*-butyllithium (*n*-BuLi) ocurred predominantly at the 3-position of **7** with formation of 2-bromo-3-lithio-1,1-dimethylindene (**10**, Scheme 3). In the absence of cycloalkanes or benzene from the hydrocarbon solvent, rather concentrated mixtures of **7** and *n*-BuLi slowly deposited unsolvated **10**, which opened the possibility of purifying **10** through simple washings with dry pentane under inert gas cover. Like the related 3-chloro-2-lithio-1,1-dimethylindene [15], **10** did not eliminate LiHal at rt, so that its final hydrolytic work-up provided clean **4** even from moderately contaminated **7**. Due to a well-known mixing problem [16–17], this final protolysis will be successful only in the absence (or at least after an adequate washing-out) of residual *n*-BuLi: Since protonation of *n*-BuLi and **10** is comparably rapid, a local depletion of the added proton source will leave the generated portion of **4** together with remnant *n*-BuLi, so that a very rapid Br/Li interchange reaction of **4** with *n*-BuLi will produce 1,1-dimethyl-2-lithioindene, whose protolysis forms 1,1-dimethylindene (**2**).

## Conclusion

*Cis*/*trans* differentiation in the saturated part of cyclopentene moieties should be based on ^1^H nuclear magnetic Overhauser enhancements rather than on the magnitudes of vicinal *cis* and *trans* ^3^*J*_H,H_ NMR coupling constants which are not always reliable. The deceivingly high value of 8.4 Hz in *trans*-2,3-dibromo-1,1-dimethylindane (*trans*-**1**) is explained in this work by a preferentially populated envelope conformation with pseudoequatorial 2-Br and hence pseudodiaxial 2-H and 3-H. The alternative (incorrect) stereoassignment (“*cis*-**19**” in reference [8] and “*cis*-**33**” in reference [7] for the presently analyzed *trans*-**1**) would have misguided us to claim erroneously that we discovered a most unusual, highly *cis* selective olefin bromination.

What else may appear unusual with **1** and its congeners? *Trans*-**1** and *cis*-**1** undergo regiospecific HBr eliminations even in the absence of bases, *cis*-**1** does so more rapidly than *trans*-**1**. The products **3** and **4**, respectively, can serve as precursors for the same product 2,3-dibromo-1,1-dimethylindene (**7**). Exploiting the unusually high inclination of 3,3-di- (**8**) and 2,3,3-tribromo-1,1-dimethylindane (**5**) toward “base-free” HBr elimination at close to rt [12], we were able to convert 1,1-dimethylindane (**9**) with NBS directly to **7**. The preparation of clean 2-bromo-1,1-dimethylindene (**4**) became possible through purification and hydrolysis of crystalline 2-bromo-3-lithio-1,1-dimethylindene (**10**).

## Experimental

**General remark. **^1^H and ^13^C NMR chemical shifts δ (ppm) were referenced to internal tetramethylsilane.

**2,3-Dibromo-1,1-dimethylindane (1)**. a) *Trans*-**1**: Tetraethylammonium bromide (18.32 g, 87.2 mmol) was dissolved with magnetic stirring in a minimum volume of chloroform (120 mL), treated with 1,1-dimethylindene (**2**, 12.55 g, 87.1 mmol, see Supporting Information File 1), and then cooled in an ice-bath. Elemental bromine (4.44 mL, 13.9 g, 87.1 mmol) in chloroform (40 mL) was added dropwise at such a rate that each drop was quickly decolorized (65 min). After warm-up to rt within the next hour, the mixture was immediately shaken with aqueous Na_2_CO_3_ (2 M) until alkaline, washed with distilled water until neutral, dried over Na_2_SO_4_, and concentrated. The crude material (23.1 g, 87%) was a colorless, liquid mixture of *trans*-**1** and *cis*-**1** (9:1) with bp 145–149 °C/11 Torr (no data given in reference [7]).

^1^H NMR (CDCl_3_, 400 MHz) δ 1.22 (s, 3H, pseudoaxial 1-CH_3_), 1.42 (s, 3H, pseudoequatorial 1-CH_3_), 4.35 (d, ^3^*J* = 8.4 Hz, 1H, 2-H), 5.40 (dd, ^3^*J* = 8.4 Hz, ^4^*J* = 0.7 Hz, 1H, 3-H), 7.17 (m, 1H, 7-H), 7.28 (m, 1H, 5-H), 7.31 (m, 1H, 6-H), 7.41 (m, 1H, 4-H) ppm, assigned through HSQC, HMBC (see below), and the following NOESY correlations: 2-H ↔ pseudoequatorial 1-CH_3_ ↔ 7-H ↔ pseudoaxial 1-CH_3_ ↔ 2-H (very weak), 4-H ↔ pseudoaxial 3-H ↔ pseudoaxial 1-CH_3_ (strong); ^1^H NMR (D_3_C–C≡N, 200 MHz) δ 1.21 and 1.43 (2 s, 2 × 3H, 2 × 1-CH_3_), 4.45 and 5.58 (AB system, ^3^*J* = 8.4 Hz, 2 × 1H, 2-H and 3-H), ca. 7.2 (m, 7-H) ppm; ^13^C NMR (CDCl_3_, 100.6 MHz) δ 25.77 (pseudoequatorial 1-CH_3_), 26.98 (pseudoaxial 1-CH_3_), 46.95 (C-1), 56.61 (C-3), 68.31 (C-2), 122.16 (C-7), 125.85 (C-4), 127.94 (C-5), 129.43 (C-6), 138.70 (C-3a), 147.68 (C-7a) ppm, assigned through HSQC and the following ^1^H/^13^C HMBC ^3^*J* and ^2^*J* interactions. ^3^*J*: 2-H → both 1-*C*H_3_, pseudoaxial 3-H → neither C-7a nor C-1, 4-H → C-6, 7-H → C-5, both 1-C*H*_3_ → C-2 and C-7a, C-3a → 5-H and 7-H, C-4 → 6-H, C-7 → 5-H, C-7a → 4-H and 6-H, pseudoaxial 1-C*H*_3_ → pseudoequatorial 1-*C*H_3_, pseudoequatorial 1-C*H*_3_ → pseudoaxial 1-*C*H_3_; ^2^*J*: 2-H → C-3, 3-H → C-2 and C-3a, both 1-C*H*_3_ → C-1. Anal. calcd for C_11_H_12_Br_2_ (304.02): C, 43.46; H, 3.98; found: C, 44.06; H, 3.92.

b) *Cis*-**1**: This was analyzed in the 3:7 mixture with *trans*-**1** as obtained from 1,1-dimethylindene (**2**) through titration with elemental bromine (not Br_3_^−^). ^1^H NMR (CDCl_3_, 400 MHz) δ 1.33 and 1.42 (2 s, 2 × 3H, 2 × 1-CH_3_), 4.34 (d, ^3^*J* = 6.1 Hz, 1H, 2-H), 5.55 (d, ^3^*J* = 6.1 Hz, 1H, 3-H), 7.20 (m, 1H, 7-H), 7.27 (m, 5-H), 7.34 (m, 6-H), 7.42 (m, 4-H) ppm, assigned through HSQC, HMBC (see below), and the following NOESY correlations: 2-H ↔ both 1-CH_3_ ↔ 7-H, but pseudoequatorial 3-H ↔ 4-H only (not 1-CH_3_); ^1^H NMR (D_3_C–C≡N, 200 MHz) δ 4.54 and 5.76 (AB system, ^3^*J* = 6.0 Hz, 2 × 1H, 2-H and 3-H) ppm; ^13^C NMR (CDCl_3_, 100.6 MHz) δ 26.07 and 26.58 (2 × 1-CH_3_), 47.23 (C-1), 55.70 (C-3), 62.07 (C-2), 123.00 (C-7), 125.56 (C-4), 127.83 (C-5), 130.00 (C-6), 139.30 (C-3a), 148.67 (C-7a) ppm, assigned through HSQC and the following ^1^H/^13^C HMBC ^3^*J* and ^2^*J* interactions. ^3^*J*: 2-H → both 1-*C*H_3_, pseudoequatorial 3-H → C-1 (strong) and C-7a (strong), both 1-C*H*_3_ → C-2 and C-7a, C-3a → 5-H and 7-H, C-4 → 6-H, C-5 → 7-H, C-6 → 4-H, C-7 → 5-H, C-7a → 4-H and 6-H, uncertain pseudoaxial 1-C*H*_3_ → pseudoequatorial 1-*C*H_3_, uncertain pseudoequatorial 1-C*H*_3_ → pseudoaxial 1-*C*H_3_; ^2^*J*: 3-H → C-3a but not C-2, both 1-C*H*_3_ → C-1; MS (EI) *m*/*z* (%): 225.008 (52, C_11_H_12_^81^Br^+^, M – Br^−^), 223.006 (62, C_11_H_12_^79^Br^+^, M – Br^−^); HRMS (EI) *m*/*z*: 223.0164 (C_11_H_12_^79^Br^+^, M – Br^−^, calcd 223.0117), no M^+^ peak.

**3-Bromo-1,1-dimethylindene (3).** A crude sample of the 9:1 mixture of *trans*-**1** and *cis*-**1** (23.0 g, max. 75 mmol) was added to a saturated solution of solid KOH (30.0 g, 833 mmol) in ethanol (190 mL) and heated to 50 °C for 4 hours. After cautious removal of some ethanol (ca. 130 mL) in vacuo, the residue was poured into distilled water (ca. 400 mL) and extracted with Et_2_O (4 × 100 mL). The combined Et_2_O extracts were washed with distilled water until neutral, dried over CaCl_2_, concentrated, and distilled to yield a pure 92:8 mixture (11.19 g, ≥67%) of 3- and 2-bromo-1,1-dimethylindene (**3** and **4**) with bp 115–117 °C/12 Torr.

^1^H NMR of **3** (CDCl_3_, 400 MHz) δ 1.32 (s, 6H, 2 × 1-CH_3_), 6.46 (s, 1H, 2-H), 7.25 (tm, ^3^*J* = 7 Hz, 1H, 6-H), 7.28 (m, 1H, 7-H), 7.30 (m, 1H, 5-H), 7.33 (dm, ^3^*J* = 7.5 Hz, 1H, 4-H) ppm, assigned through HSQC and ^1^H/^13^C HMBC (see below); ^13^C NMR (CDCl_3_, 100.6 MHz) δ 24.17 (2 × 1-CH_3_), 49.97 (C-1), 118.19 (C-3), 120.48 (C-4), 120.81 (C-7), 126.41 (C-6), 126.87 (C-5), 140.84 (C-3a), 145.13 (C-2), 151.74 (C-7a) ppm, assigned through HSQC and the following ^1^H/^13^C HMBC ^3^*J* interactions. 1-C*H*_3_ → C-2 and C-7a, 2-H → C-3a and C-7a, C-3 → 4-H → C-6, C-3a → 7-H → C-5, C-4 → 6-H, C-7 → 5-H, C-7a → 2-/4-/6-H; Anal. calcd for C_11_H_11_Br (223.13): C, 59.22; H, 4.97; found [18]: C, 58.79,; H, 4.91. The alternative HBr elimination with KO*t*-Bu was rapid and exothermic in diglyme as the solvent.

**2-Bromo-1,1-dimethylindene (4).** A sample of contaminated 2,3-dibromo-1,1-dimethylindene (**7**, 8.43 g, 27.7 mmol) in pentane (5.0 mL) was treated with *n*-BuLi (ca. 40 mmol) in hexane (35 mL) at −70 °C under argon gas cover. Since this reaction was run in an acyclic, saturated hydrocarbon as the solvent (no cyclopentane, no benzene), a voluminous precipitate began to emerge slowly at rt within ca. one hour. When this colorless powder of 2-bromo-3-lithio-1,1-dimethylindene (**10**) had settled after 20 hours at rt, the supernatant was withdrawn by syringe, and the residue was washed with dry pentane (3 × 15 mL). This purified, solid material was suspended in pentane at −70 °C under argon gas and quenched with methanol (2.0 mL). After dilution with water and Et_2_O, the aqueous layer was extracted with Et_2_O (2×). The combined Et_2_O phases were washed with water until neutral and dried over Na_2_SO_4_. The crude material contained **4** and 1,1-dimethylindene (**2**, 77:23) without any other indene derivative. Pure **4** (2.10 g, 34%) distilled at 101–103 °C/12 Torr. ^1^H NMR (CDCl_3_, 400 MHz) δ 1.25 (s, 6H, 2 × 1-CH_3_), 6.75 (s, 1H, 3-H), 7.15 (tm, ^3^*J* = 7.5 Hz, 1H, 6-H), 7.18 (tm, ^3^*J* = 7.5 Hz, 1H, 5-H), 7.23 (dm, ^3^*J* = 7.5 Hz, 1H, 4-H), 7.27 (dm, ^3^*J* = ca. 7 Hz, 1H, 7-H) ppm, assigned through comparisons with **3** and **7**, HSQC and ^1^H/^13^C HMBC (see below), and the NOESY correlation 1-CH_3_ ↔ 7-H; ^1^H NMR (D_3_C–C≡N, 200 MHz) δ 1.25 (s, 6H, 2 × 1-CH_3_), 6.89 (s, 1H, 3-H) ppm; ^13^C NMR (CDCl_3_, 100.6 MHz) δ 23.94 (2 × 1-CH_3_), 52.05 (C-1), 120.48 (C-4), 121.51 (C-7), 125.11 (C-6), 126.70 (C-5), 128.99 (C-3), 139.86 (C-2), 141.07 (C-3a), 151.66 (C-7a) ppm, assigned through HSQC, comparison with **7**, and the following ^1^H/^13^C HMBC ^3^*J* and ^2^*J* interactions. ^3^*J*: 1-C*H*_3_ → 1-*C*H_3_, 1-C*H*_3_ → C-2 and C-7a, 3-H → C-1 and C-7a, 7-H → C-3a and C-5, C-6 → 4-H; ^2^*J*: 1-C*H*_3_ → C-1, 3-H → C-3a; MS (EI, 70 eV, 80 °C) *m*/*z* (%) 224 and 222 (2 × 11, M^+^), 143 (100, M – Br^−^), 128 (56, M^+^ − CH_3_Br); HRMS (EI) *m*/*z* 224.0026 (C_11_H_11_^81^Br^+^, M^+^), 222.0045 (C_11_H_11_^79^Br^+^, M^+^); Anal. calcd for C_11_H_11_Br (223.13): C, 59.22; H, 4.97; found: C, 60.55 H, 4.97.

**2,3,3-Tribromo-1,1-dimethylindane (5):** This thermolabile adduct of 3-bromo-1,1-dimethylindene (**3**) and elemental bromine (see **7**) was not purified but recognized through its ^1^H NMR chemical shifts and its thermolysis product **7**. ^1^H NMR (CDCl_3_, 200 MHz) δ 1.36 (s, 3H, 1-CH_3_), 1.39 (s, 3H, 1-CH_3_), 4.78 (s, 1H, 2-H) ppm; ^1^H NMR (CCl_4_, 200 MHz) δ 1.34 (s, 3H, 1-CH_3_), 1.37 (s, 3H, 1-CH_3_), 4.69 (s, 1H, 2-H) ppm.

**2,2,3-Tribromo-1,1-dimethylindane (6):** A crude sample of 2-bromo-1,1-dimethylindene (**4**, ca. 3 mmol) in CCl_4_ (4 mL) was overtitrated with elemental bromine in CCl_4_ solution at rt. After 3 hours, the excess of bromine was swept off in a stream of N_2_ gas or destroyed with aqueous sodium sulfite. Almost pure **6** distilled at 157–161 °C (bath temp.)/3 mbar as a nearly colorless, viscos liquid (528 mg, 1.38 mmol). ^1^H NMR (CDCl_3_, 400 MHz) δ 1.48 (s, 3H, pseudoaxial 1-CH_3_), 1.72 (s, 3H, pseudoequatorial 1-CH_3_), 5.94 (s, 1H, 3-H), 7.18 (dm, ^3^*J* = 7.4 Hz, 1H, 7-H), 7.31 (td, ^3^*J* = 7.4 Hz, ^4^*J* = 1.3 Hz, 1H, 5-H), 7.35 (td, ^3^*J* = 7.2 Hz, 1H, 6-H), 7.42 (dm, ^3^*J* = 7 Hz, 1H, 4-H) ppm, assigned through HMBC (see below) and the NOESY correlations 4-H ↔ pseudoaxial 3-H ↔ pseudoaxial 1-CH_3_ ↔ 7-H ↔ pseudoequatorial 1-CH_3_; ^13^C NMR (CDCl_3_, 100.6 MHz) δ 27.49 (slighly broadened pseudoequatorial 1-CH_3_), 27.84 (pseudoaxial 1-CH_3_), 55.18 (C-1), 63.97 (C-3), 84.42 (C-2), 122.45 (C-7), 125.64 (C-4), 128.03 (C-5), 129.77 (C-6), 137.56 (C-3a), 146.18 (C-7a) ppm, assigned through HSQC and the following ^3^*J* and ^2^*J* HMBC cross peaks. ^3^*J*: both 1-C*H*_3_ → C-2 and C-7a, 3-H → C-4 and C-7a (but not C-1 since 3-H is pseudoaxial), 4-H → C-6 and C-7a, 7-H → C-3a and C-5, C-4 → 6-H, C-3a → 5-H; ^2^*J*: both 1-C*H*_3_ → C-1, 3-H → C-3a; HRMS and MS (EI) *m*/*z* (%) 385.8143 (0.1, C_11_H_11_^81^Br_3_^+^, M^+^), 383.8302 (0.4, C_11_H_11_^79^Br^81^Br_2_^+^, M^+^), 381.8358 (0.5, C_11_H_11_^79^Br_2_^81^Br^+^, M^+^), 379.8274 (0.3, C_11_H_11_^79^Br_3_^+^, M^+^), 304.98 (12, C_11_H_11_^81^Br_2_^+^, M – Br^−^), 302.98 (26, C_11_H_11_^79^Br^81^Br^+^, M – Br^−^), 300.98 (13, C_11_H_11_^79^Br_2_^+^, M – Br^−^).

**2,3-Dibromo-1,1-dimethylindene (7):** a) From 3-bromo-1,1-dimethylindene (**3**): A solution of **3** (4.08 g, 18.3 mmol) in CCl_4_ (10 mL) was cooled and stirred in an ice-bath and titrated with elemental bromine (2.92 g, 18.3 mmol) in CCl_4_. The initially instantaneous decolorization of bromine became progressively slower with a half-reaction time of roughly 2 min toward the end. After 2 hours at rt, the solution contained some product **7** and mainly 2,3,3-tribromo-1,1-dimethylindane (**5**). Upon CCl_4_ evaporation in vacuo up to 80 °C (darkening), the primary product **5** became thermally converted into **7** during distillation that yielded **7** (4.42 g, 80%) as a colorless, slightly light-sensitive liquid; analytically pure **7** had bp 144–146 °C/13 Torr. ^1^H NMR (CDCl_3_, 400 MHz) δ 1.31 (s, 6H, 2 × 1-CH_3_), 7.26 (m, ^3^*J* = 7.5 Hz, 1H, 6-H), 7.30 (m, 1H, 5-H), 7.31 (m, 1H, 7-H), 7.36 (dm, ^3^*J* = 7.5 Hz, 1H, 4-H) ppm, assigned through HSQC, comparison with **3**, and the NOESY correlation 1-CH_3_ ↔ 7-H; ^13^C NMR (CDCl_3_, 100.6 MHz) δ 24.2 (2 × 1-CH_3_), 52.6 (C-1), 120.0 (C-3), 120.3 (C-4), 121.3 (C-7), 126.4 (C-6), 127.2 (C-5), 138.9 (C-2), 139.9 (C-3a), 150.0 (C-7a) ppm, assigned through HSQC, comparison with **3**, and the following ^3^*J* and ^2^*J* HMBC cross peaks. ^3^*J*: 1-C*H*_3_ → C-2 and C-7a, 4-H → C-3 and C-6 and C-7a, 7-H → C-3a and C-5, C-4 → 6-H, C-7 → 5-H; ^2^*J*: 1-C*H*_3_ → C-1; HRMS (EI) *m*/*z* (%) 303.9135 (14, C_11_H_10_^81^Br_2_^+^, calcd 303.9103, M^+^), 301.9119 (25, C_11_H_10_^79^Br^81^Br^+^, calcd 301.9123, M^+^), 299.9146 (14, C_11_H_10_^79^Br_2_^+^, calcd 299.9144, M^+^), 222.9936 (83, C_11_H_10_^81^Br^+^, M – Br^−^), 220.9974 (77, C_11_H_10_^79^Br^+^, M – Br^−^); Anal. calcd for C_11_H_10_Br_2_ (302.0): C, 43.75; H, 3.34; Br, 52.92; found: C, 44.06; H, 3.42; Br, 52.00.

b) From 2-bromo-1,1-dimethylindene (**4**) via 2,2,3-tribromo-1,1-dimethylindane (**6**): A small sample (44 mg, 0.11 mmol) of distilled tribromide **6** (obtained as above from **4** and containing no trace of **7**) in CCl_4_ was placed in an NMR tube (5 mm) and treated with an excess of solid KO*t*-Bu, which consumed **6** within less than 2 hours at rt. Aqueous work-up with Et_2_O afforded the dibromide **7** (29 mg, 87%) as the only product (hence, no S_N_2 reaction by KO*t*-Bu). NEt_3_ as the base in place of KO*t*-Bu required 12 days at rt.

c) From 1,1-dimethylindane (**9**): A mixture of *N*-bromosuccinimide (NBS, 7.13 g, 40 mmol), 1,1-dimethylindane (**9**, 1.46 g, 10 mmol, see Supporting Information File 1), and CCl_4_ (50 mL) was treated with azobis(isobutyronitrile) (40 mg) and warmed slowly up to 85 °C. After 30 min of vived refluxing, the dark red suspension showed traces of elemental bromine in the gas phase and was cooled in an ice bath (15 min). A ^1^H NMR spectrum of the solution revealed that the starting material **9** was completely consumed and that a mixture containing three gem-dimethyl compounds had been generated: 2,3-dibromo-1,1-dimethylindene (**7**, 76%), **1** (1%, *trans*/*cis* ca. 3:2), and an unknown side-product (20%). The suspension was filtered, and the undissolved portion was washed with CCl_4_ (2 × 5 mL), affording a colorless, powdery mixture of NBS and succinimide (4:36 by ^1^H NMR). The dark red CCl_4_ filtrate became colorless on shaking with an aqueous solution of sodium sulfite (at least 0.6 g) and was washed with distilled water (10 mL), then dried through stirring with granulated CaCl_2_ (30 min). After removal of CaCl_2_, the colorless solution became violet on stirring with a sufficient amount of solid KO*t*-Bu (2.6 g) for 30 min, which destroyed the two dibromides **1** and other small contaminations. The CCl_4_ solution was shaken with H_2_O (10 mL), aqueous HCl (2 M, 10 mL), and H_2_O until neutral, then dried as above with CaCl_2_ (35 min). Evaporation of CCl_4_ and subsequent distillation in vacuo furnished the colorless liquid **7** (1.35 g, 45%).

## Supporting Information

File 1 Alternative synthetic routes to 1,1-dimethylindene (**2**) and congeners; experimental procedures for **2**, 1,1-dimehylindane (**9**), 3-methyl-1-phenylbutan-2-ol, *N*-(1,1-dimethylindan-3-ylidene)hydrazine, and *N,N´*-bis(1,1-dimethylindan-3-ylidene)hydrazine.
