# Supplementary material for: Unusual traits of cis and trans-2,3-dibromo-1,1-dimethylindane on the way from 1,1-dimethylindene to 2-bromo-, 3-bromo-, and 2,3-dibromo-1,1-dimethylindene
Source: Beilstein J Org Chem. 2016 Jun 10;12:1178–84. doi: 10.3762/bjoc.12.113 (PMC4979680; doi:10.3762/bjoc.12.113)
Supplement: File 1 — Alternative synthetic routes to 1,1-dimethylindene (2) and congeners; experimental procedures for 2, 1,1-dimehylindane (9), 3-methyl-1-phenylbutan-2-ol, N-(1,1-dimethylindan-3-ylidene)hydrazine, and N,N´-bis(1,1-dimethylindan-3-ylidene)hydrazine. [file Beilstein_J_Org_Chem-12-1178-s001.pdf]

Supporting Information

for

Unusual traits of *cis* and *trans*-2,3-dibromo-1,1-dimethylindane on the way from 1,1-dimethylindene to 2-bromo-, 3-bromo-, and 2,3-dibromo-1,1-dimethylindene

Rudolf Knorr*, David S. Stephenson, Ernst Lattke, Petra Böhrer, and Jakob Ruhdorfer

Address: Department Chemie, Ludwig-Maximilians-Universität München, Butenandtstrasse 5–13 (Haus F), 81377 München, Germany

Email: Rudolf Knorr* - rhk@cup.uni-muenchen.de

* Corresponding author

Alternative synthetic routes to 1,1-dimethylindene (2) and congeners; experimental procedures for 2, 1,1-dimehylindane (9), 3-methyl-1-phenylbutan-2-ol, *N*-(1,1-dimethylindan-3-ylidene)hydrazine, and *N*,*N*´-bis(1,1-dimethylindan-3-ylidene)hydrazine

Alternative synthetic routes to 1,1-dimethylindene (2) and congeners

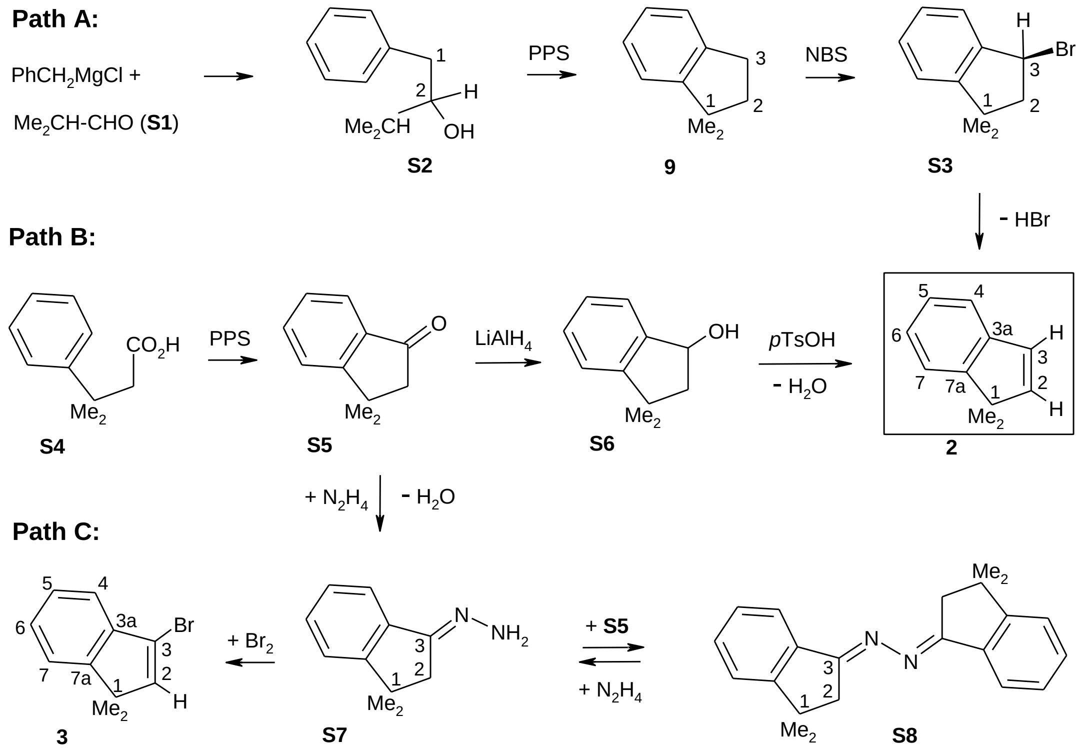

Scheme S1: Pathways A and B to olefin **2** and pathway C to 3-bromo-1,1-dimethylindene (**3**); NBS = *N*-bromosuccinimide, PPS = polyphosphoric acid, *p*TsOH = *p*-toluenesulfonic acid.

The method of Bogert and Davidson [S1] (path A in Scheme S1) furnished the alcohol **S2**, whose cyclization to give 1,1-dimethylindane (**9**) was performed with polyphosphoric acid (PPS) in place of conc. H_2SO_4 (90% [S1]) so as to avoid any sulfonation of the aromatic ring. Monobromination of **9** with *N*-bromosuccinimide (NBS, 1 equiv or less) and the radical starter azobis(isobutyronitrile) (AIBN) afforded the hitherto unknown 3-bromo-1,1-dimethylindane (**S3**), which decomposed slowly above 110 °C with elimination of gaseous HBr and darkening. Therefore, slow distillation under a pressure of not less than 49 Torr was required to secure olefin **2** as the most volatile hydrocarbon. However, overbromination by NBS could not entirely be avoided even with a substoichiometric amount of NBS, so that the thermal HBr elimination generated some 3-bromoalkene **3** (shown in the lower left corner of Scheme S1) which appeared in the higher-boiling fractions of the distilled product.

The alternative procedure of Bosch and Brown [S2] (path B in Scheme S1) for preparing the olefin **2** can also be recommended if the dehydration of 1,1-dimethylindan-3-ol (**S6**) is carried out by distillation over 4-toluenesulfonic acid in place of oxalic acid or instead of an ester pyrolysis

[S3]. As already communicated [S4], the precursor acid **S4** was conveniently cyclized with PPS to afford 1,1-dimethylindan-3-one (**S5**) [S5]. This ketone was a productive precursor of **S6**; but it afforded only poor yields of 1,1-dimethylindane (**9**) under the usual Clemmensen or Wolff–Kishner conditions. In the latter attempt, the major portion of **S5** and hydrazine hydrate produced the azine **S8**. This evasive behavior may have thwarted [S6] a proper characterization of the liquid hydazone **S7** which also forms **S8** on distillation. Therefore, we reconverted the azine **S8** to the crude hydrazone **S7** through heating with hydrazine hydrate (path C). The subsequent treatment of **S7** with elemental bromine in pyridine and Et_2O furnished 3-bromo-1,1-dimethylindene (**3**) [S4] in modest but variable yields.

Experimental procedures

1,1-Dimethylindene (2) from 9 via S3 (path A). 1,1-Dimethylindane (**9**, 42.5 g, 291 mmol), *N*-bromosuccinimide (NBS, 51.86 g, 291 mmol), dry CCl_4 (600 mL), and azobis(isobutyronitrile) (AIBN, 100 mg) were placed in a round-bottomed flask fitted with a reflux condenser. Slow heating initiated violent boiling with formation of colorless succinimide. After final weak refluxing for 30 min, all NBS (the solid bottom phase) had been consumed. Upon cooling to rt, succinimide floated on the surface and was collected on a filter, then washed with CCl_4 (3×). The combined CCl_4 filtrates were concentrated in vacuo, and the residual 3-bromo-1,1-dimethylindane (**S3**), containing traces of the olefin **2** and the bromoalkene **3**, was characterized by its ^{1}H NMR spectrum ($CDCl_3$, 200 MHz): δ 1.22 and 1.42 (2 s, 2 × 3H, 2 × 1-CH_3), ca. 2.39 and 2.51 (AB part of an ABX system, ^2J = 14.2 Hz, ^3J = 4.1 and 7.0 Hz, 2H, 2 × 2-H), 5.48 (dd, X part, apparent ^3J = 6.1 and 4.1 Hz, 1H, 3-H), 7.10 (m, 2H), 7.20 (m, 1H), 7.32 (m, 1H) ppm.

Purification of **S3** by distillation was not feasible because of thermal HBr elimination that gave the olefin **2**. Therefore, **2** was generated from this crude material through slow distillation: Yield 35 g (83%); bp 105–106 °C/49 Torr (reference [S2]: 57 °C/4.8 Torr; reference [S3]: 190–192 °C); ^{1}H NMR (CCl_4, 220 MHz) δ 1.27 (s, 6H, 2 × 1-CH_3), 6.24 (d, ^3J = 5.5 Hz, 1H, 2-H), 6.53 (d, ^3J = 5.5 Hz, 1H, 3-H), 7.07 (m, 2H), 7.16 (m, 2H) ppm. The higher-boiling fractions contained a small amount of 3-bromo-1,1-dimethylindene (**3**).

1,1-Dimethylindane (9). The alcohol **S2** (3.90 g, 23.7 mmol) and polyphosphoric acid [20.2 mL of a mixture of phosphoric acid (85%, 100 mL) and P_2O_5 (210 g)] was warmed to 75 °C and stirred for 30 min. The biphasic product mixture was treated with ice-water and Et_2O, and the separated aqueous layer was shaken with Et_2O (2×). The combined Et_2O layers were shaken with water (1×), aqueous NaOH (2 M, 1×), then washed with ice-water until neutral, dried over

Na_2SO_4, concentrated and distilled, affording pure 1,1-dimethylindane (**9**, 2.62 g, 67%) with bp 110–155 °C (bath temp.)/50 Torr (reference [S1]: 191 °C). ^{1}H NMR ($CDCl_3$, 200 MHz) δ 1.25 (s, 6H, 2 × CH_3), 1.91 (t, ^3J = 7.2 Hz, 2H, 2 × 2-H), 2.88 (t, ^3J = 7.2 Hz, 2H, 2 × 3-H), 7.14 (quasi-s, 4H, C_6H_4) ppm; IR (film) ν 2958, 1479, 756 cm^{-1}. Anal. calcd for $C_{11}H_{14}$ (146.2): C, 90.35; H, 9.65. Found: C, 90.40; H, 9.76.

3-Methyl-1-phenylbutan-2-ol (S2). A dry three-necked, round-bottomed flask (1 L) was fitted with a large pressure-equalizing dropping funnel and a double-walled reflux condenser carrying a drying tube. The flask was charged with magnesium turnings (19.7 g, 807 mmol), a strong magnetic stirring bar, and anhydrous diethyl ether (25 mL). Without stirring, the Grignard reaction was started through the dropwise addition of ca. 20 mL of the solution of benzyl chloride (102.1 g, 807 mmol) in anhydrous Et_2O (400 mL) and weak warming of the bottom layer of magnesium metal. As soon as signs of local boiling became visible, the stirrer was started and the remaining benzyl chloride solution was added dropwise at a rate (within 4 hours) which kept the Et_2O slightly boiling without external warming. This precaution served to prevent the formation of 1,2-diphenylethane and was successful since almost all of the Mg turnings had been consumed after further heating to reflux for 30 min. Without heating, a solution of 2-methylpropanal (**S1**, 58.3 g, 808 mmol) in anhydrous Et_2O (100 mL) was added dropwise to the vigorously stirred mixture within 150 min. After short final heating to reflux (30 min), the slow addition of distilled water (50 mL within 90 min) to the cooled and stirred mixture produced a white precipitate. The product-containing supernatant was decanted and set aside, while the precipitate was dissolved with aqueous H_2SO_4 (2 M, 400 mL), then shaken together with the supernatant in a big separation funnel (2 L), whereafter the separated (lower) aqueous layer was discarded. The Et_2O phase was shaken with water (3×), then aqueous NaOH (2 M, 2×), then water until neutral, and dried over K_2CO_3. Evaporation and distillation furnished the product alcohol **S2** (55.3 g, 42%) with bp 112–113 °C/14 Torr (reference [S1]: 118–122 °C/14 Torr). ^{1}H NMR (CCl_4, 60 MHz) δ 0.92 (d, ^3J = 6.2 Hz, 6H, 2 × CH_3), 1.55 (m, 1H, 3-H), 1.68 (broad s, 1H, OH), 2.52 and 2.65 (AB part of an ABX system, 2H, CH_2-1), 3.37 (dt, 1H, 2-H), 7.10 (quasi-s, 5H, C_6H_5) ppm.

***N*-(1,1-Dimethylindan-3-ylidene)hydrazine (S7).** A suspension of the azine **S8** (7.28 g, 23.0 mmol) and dry hydrazine hydrate (1.20 mL, 1.23 g, 24.7 mmol) in propan-1-ol (20 mL) was refluxed for 30 hours, affording a homogeneous, yellow solution. Propan-1-ol was removed in a rotary evaporator, whereupon the pot residue was diluted with cold cyclohexane (20 mL). The precipitating remnant azine **S8** was separated by filtration, and the filtrate was concentrated at rt

to yield the crude hydrazone **S7** as an almost colorless, viscous liquid (7.16 g, 90%). ^{1}H NMR (CCl_4, 60 MHz) δ 1.30 (s, 6H, 2 × CH_3), 2.42 (s, 2H, CH_2), 5.30 (broad s, NH_2), 7.23 (m, 3H, 5-/6-/7-H), 7.63 (m, 1H, 4-H) ppm. This material furnished the known [S6] 3-iodo-1,1-dimethyl-indene on treatment with elemental iodine and NEt_3 or gave **3** with elemental bromine in pyridine.

***N,N´*-Bis(1,1-dimethylindan-3-ylidene)hydrazine (S8).** A mixture of ethanol (30 mL), 1,1-dimethylindan-3-one (**S5**, 13.86 g, 86.7 mmol), and hydrazine hydrate (27.7 g, 554 mmol) was heated at 100 °C for 8 hours. Ethanol and unconsumed hydrazine hydrate were distilled off under 12 Torr at bath temperatures up to 140 °C, whereupon the residue was crystallized from methanol (20 mL) to yield shiny yellow platelets of the azine **S8** (5.73 g, 42% after repeated concentration steps). Mp 164–165 °C (methanol or cyclohexane); ^{1}H NMR ($CDCl_3$ or CCl_4, 60 MHz) δ 1.38 (s, 12H, 4 × CH_3), 2.97 (s, 4H, 2 × CH_2), 7.40 (m, 6H, 2 × 5-/6-/7-H), 7.93 (m, 2H, 2 × 4-H) ppm; IR (KBr) ν 3090, 3068, 3022, 2965, 2920, 2865, 1638 (C=N), 1605, 1468, 1321, 762 cm^{-1}. Anal. calcd for $C_{22}H_{24}N_2$ (316.4): C, 83.50; H, 7.65; N, 8.85. Found: C, 83.70; H, 7.73; N, 8.70..

References

S1. Bogert, M. T.; Davidson, D. *J. Am. Chem. Soc.* **1934**, *56*, 185–190.

S2. Bosch, A.; Brown, R. K. *Can. J. Chem.* **1964**, *42*, 1718–1733.

S3. Jönsson, N. A.; Kempe, T.; Mikiver, L.; Gahlin, K.; Sparf, B. *Acta Pharm. Suecica* **1981**, *18*, 349–372; *Chem. Abstr.* **1982**, *96*, 122365a. Compound **25** on pp 360–361 therein.

S4. von Roman, U.; Ruhdorfer, J.; Knorr, R. *Synthesis* **1993**, 985–992.

S5. Protocol for compound **11i** on p 990 in reference [S4] (read “3-” instead of “2-”Bromo!).

S6. Bunnett, J. F.; Creary, X.; Sundberg, J. E. *J. Org. Chem.* **1976**, *41*, 1707–1709.
